# First survey on seroprevalence of Japanese encephalitis in long-tailed macaques (*Macaca fascicularis*) in Bali, Indonesia

**DOI:** 10.14202/vetworld.2022.1341-1346

**Published:** 2022-05-27

**Authors:** I Gusti Agung Arta Putra, Anak Agung Ayu Mirah Adi, I Nyoman Mantik Astawa, I Made Kardena, I Nengah Wandia, I Gede Soma, Fany Brotcorne, Agustin Fuentes

**Affiliations:** 1 Laboratory of Animal Anatomy and Physiology, Faculty of Animal Husbandry, Udayana University, Kampus Bukit, Jimbaran, Badung, Bali, Indonesia; 2 Primate Research Center, Udayana University, Kampus Bukit, Jimbaran, Badung, Bali, Indonesia; 3 Laboratory of Veterinary Pathology, Faculty of Veterinary Medicine, Udayana University, Kampus Sudirman, Jalan PB Sudirman, Denpasar, Bali, Indonesia; 4 Laboratory of Veterinary Virology, Faculty of Veterinary Medicine, Udayana University, Kampus Sudirman, Jalan PB Sudirman, Denpasar, Bali, Indonesia; 5 Research Unit SPHERES, Department of Biology, Ecology, and Evolution, University of Liege, Belgium; 6 Department of Anthropology, Princeton University, 123 Aaron Burr Hall, Princeton NJ 08544, United States

**Keywords:** enzyme-linked immunosorbent assay, Japanese encephalitis virus, *Macaca fascicularis*, seroprevalence

## Abstract

**Background and Aim:**

Japanese encephalitis (JE) is a zoonotic infectious inflammatory brain disease caused by the JE virus (JEV). Considerable research into the seroprevalence of JE in domestic animals has been conducted, but there have been no reports of its occurrence in wild animals, including long-tailed macaques (*Macaca fascicularis*). This study aimed to estimate the seroprevalence of JEV infection and its determinants in long-tailed macaques in Bali and the prevalence of mosquito vectors.

**Materials and Methods:**

Blood samples (3 mL) were collected from a population of *M. fascicularis* (92 heads) inhabiting a small forest with irrigated rice field nearby (wetland area) in Ubud, Gianyar, and from two populations in dryland areas with no wet rice field (Uluwatu, Badung, and Nusa Penida, Bali Province, Indonesia). The collected sera were tested for antibodies against JEV using a commercially available enzyme-linked immunosorbent assay kit (qualitative monkey JE Immunoglobulin G antibody kit). The seropositivity of the antibodies was then compared based on different variables, namely, habitat type, age, and sex.

**Results:**

The seroprevalence of the JEV antibodies in all the samples tested was found to be 41.3%. The seropositivity of the monkey serum samples collected from the wetland area was 46.4%, which was higher than the seropositivity of the sera samples collected from the dried field areas (1.25%). Monkey sera collected from the wetland areas were 6.1 times (odds ratio [OR]: 6.1; 95% confidence interval [CI]: 0.71-51.5, p>0.05) more likely to be seropositive compared to the monkey sera collected from the dried field areas. Meanwhile, female monkeys were 1.79 times (OR: 1.79; 95% CI: 0.76-4.21; p>0.05) more likely to be seropositive to JEV than males. Similarly, juvenile monkeys were 2.38 times (OR: 2.38; 95% CI: 0.98-5.79); p>0.05) more likely to be seropositive against the JEV than adult monkeys. However, none of these differences achieved statistical significance. Regarding the JEV mosquito vector collection, more *Culex* mosquitoes were found in the samples from the wetland areas than from the dried field areas.

**Conclusion:**

The study confirms the existence of JEV infection in long-tailed macaques in Bali. There were patterned seropositivity differences based on habitat, age, and sex of the monkeys, but these were not significant. The possibility of monkeys as a JEV reservoir and the presence of the mosquitoes as the JEV vector are suggested but require more study to confirm.

## Introduction

Japanese encephalitis (JE) is considered a neglected tropical disease with a significant negative impact on human and animal populations. Japanese encephalitis virus (JEV) infection does not cause clinical symptoms in most species. However, around a quarter of the infected population may show neurological symptoms. Humans are the dead-end hosts of the virus, and, in these hosts, the virus causes serious acute encephalitis [1,2]. Similar clinical symptoms have also been recorded in horses and mules [3]. In other infected animals, such as ducks, cows, sheep, and buffalo, the symptoms of encephalitis are not visible, although JEV antibodies can be detected [4]. Several livestock species in Bali demonstrate the presence of seropositive antibodies against JEV [5,6].

The presence of livestock and mosquitoes as vectors can lead to the transmission of JEV to humans. As humans increasingly move into wildlife habitats, wherein the vectors are present, the role of wildlife as transmitters of JEV needs to be considered. Antibodies seropositive to JEV in monkey species such as *Macaca fascicularis*, *Macaca fuscata*, and *Macaca nemestrina* have been reported in several Asian countries [7,8]; however, to date, Indonesia, in general, and Bali, in particular, has not reported JEV seroprevalence in monkeys. Transmission of the JEV occurs from a viremia state of the infected host to other susceptible hosts through vector bites [9]. Ardeid birds and pigs are the main reservoirs of JEV. Ardeid wading birds are the primary maintenance hosts for JEV, while pigs are the main amplifying hosts. *Culex* mosquitoes are stated to be the primary mosquito vectors [2,10-13]. In contrast, several other mosquito genera such as *Anopheles* spp., *Aedes* spp., *Armigeres* spp., and *Mansonia* spp. are also reported to be the potential JEV vectors [14]. The natural transmission cycle of JEV involves an enzootic (sylvatic) mosquito-bird-mosquito and/or mosquito-pig-mosquito cycle [15]. Five major mosquito species, namely, *Culex tritaeniorhynchus*, *Culex quinquefasciatus*, *Culex fuscocephala*, *Anopheles vagus*, and *Aedes albopictus* have been reported in the Bali Province [6].

Many researches [3-5] have been carried out on JE seroprevalence in domestic animals, but there have been few reports of its prevalence in wild animals. Moreover, the occurrence of JEV in long-tailed macaques has not yet been investigated. In this study, we screened for JEV antibodies in *M. fascicularis* in the Bali Province to determine whether they had been infected with the JEV or not. Blood samples from *M. fascicularis* inhabiting the monkey forest at Ubud, Gianyar; Uluwatu, Badung; and Nusa Penida, Bali Province in Indonesia were sampled.

This study aimed to estimate the seroprevalence of JEV infection in long-tailed macaques in Bali and to investigate the distribution of seropositivity according to habitat type (wetland area vs. dry land area), age (juvenile vs. adult), and sex.

## Materials and Methods

### Ethical approval

This study has been officially approved by the Animal Ethics Committees of Faculty of Veterinary Medicine, Udayana University with reference number 449A/UN14.2.9/PG/2019.

### Study period and areas

Sample collection in Ubud-Gianyar and Uluwatu-Badung was conducted from January to March 2019 and that in Nusa Penida was conducted in July 2019. The study area is classified into two habitat types: wetland and rocky dry areas. The wetland area, our primary study site with range of temperature, humidity, and rainfall of 22-31°C, 85-90%, and 98-350 mm, respectively, fulfills several criteria that theoretically facilitate the transmission of JEV. This study site has a large long-tailed macaque population (n=1100 individuals) [16] in a small tropical forest fragment surrounded by agricultural land, towns, and rivers, and many people visit it for tourism. There are wet rice fields nearby and the town nearby has a backyard pig farm. The forest area has an area of 12.5 hectares at an altitude of 300 m above sea level.

The secondary study site (dry land area with range of temperature, humidity, and rainfall of 26-31°C, 83%, and 24-337 mm, respectively) consists of chalky dry soils with no wet-rice agriculture. It was selected as a supplementary site to assess the role of aridity in infection rates. The site is a shrub forest and rocky dry area, called Uluwatu Temple, and is located in Badung Regency in southern tip of Bali. Uluwatu Temple is 97 m above sea level with a macaque population of around 330 individual [17]. The other dry area chosen was located on the small island, named Nusa Penida, as a karst area with range of temperature, humidity, and rainfall of 29-30°C, 85-90%, and 85-345 mm, respectively, located to the southeast of Bali, and belongs to the Klungkung Regency area of Bali Province. In this third area, there is a temple named Goa Giri Putri. The Goa Giri Putri Temple location is close to the shoreline at an altitude of 150 m from sea level. We found several groups of long-tailed macaques inhabiting both dryland sites, though no estimates of the macaque population are available.

### Blood collection and serum preparation

**Figure-1:**
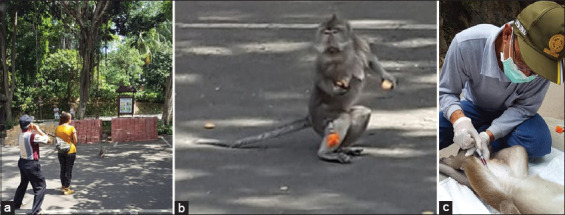
(a) Remote drug administration using blowpipe, (b) dart placed on thigh, and (c) blood collection from femoral vein following immobilization.

Depending on the study site, macaques were captured either using a combination of ketamine and xylazine (Figure-1a and b) administered remotely using darts projected through a blowpipe or were lured using food placed into trapping cages (L×W×H of 4 m×3 m×2.5 m) followed by the administration of drugs intramuscularly after squeezing the animals in smaller squeeze cages [18]. Blood (~3 mL) was collected from the femoral vein of each of the 92 sampled monkeys (Figure-1c) in plain vacutainer tubes (Zhejiang GongDong Medical Technology Co., Ltd, China). The serum was extracted and stored at −20°C at the laboratory of the Primate Research Center, Udayana University, until the enzyme-linked immunosorbent assay (ELISA) test was performed.

### Mosquito collection and identification

Mosquitoes were collected from the wetland study site (Ubud) and dryland study site (Uluwatu) using mosquito light traps (Black Tube Mosquito Trap). No mosquitoes were captured at the Nusa Penida site. Two mosquito traps were placed in a moist area and protected from the wind. The traps were set for 12 h, from 18.00 h to 06.00 h, for three consecutive days. The trapped mosquitoes were transported alive to the laboratory for species identification using descriptions and illustration keys adapted from Depkes RI [19] and with the assistance of an entomologist.

### ELISA tests

The serum samples were tested for anti-JEV antibodies using a commercial ELISA test (qualitative monkey JE Immunoglobulin G antibody kit) (Cat No: MBS 935233-MyBioSource, Inc., San Diego, CA 92195-3308, USA). The samples were tested without dilution based on the manufacturer’s instructions. The results were read using an ELISA plate reader at a wavelength of 450 nm. The optical densities (ODs) of the negative control were averaged, and the cutoff values (CoV) were determined by calculating the average of the negative control wells +0.15.

### Statistical analysis

Data obtained in the form of OD values were tabulated, and the seroprevalence was calculated by a formula:

(A/B) × 100%,

where A= number of positive samples, and B= total number of sample. To analyze the factors likely to influence infection, namely, sex, age, and the sampled habitat type, the univariate analysis odds ratio (OR) was used with a 95% confidence interval (CI). An exact test mid-*P* two-tail significance of p<0.05 was calculated using the online open-source software OpenEpi version 3.01 (www.OpenEpi.com). The number of mosquitoes collected was calculated and analyzed.

## Results

**Figure-2:**
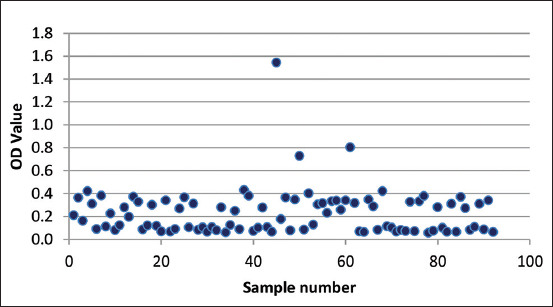
Optical density (OD) value of samples: OD value higher than cutoff values (0.262) was considered positive for JEV Immunoglobulin G.

The standard for assessing the CoV on the indirect ELISA for the JE in the animal test was established [5]. A commercial ELISA kit was used, and the test is considered to have comparable sensitivity and specificity with that of the JE gold standard tests [20]. The CoV value in these test results was averaged by the negative control value +0.15. The calculated CoV was 0.262. The profile of the OD values of the sample varied (Figure-2). Samples that had an OD value higher than the CoV (0.262) were stated as positive.

Overall, the prevalence of antibodies against JEV in long-tailed macaques in this study was 43.4%. In the wetland area, seropositivity was 46.4%, and in the dry area, seropositivity was 12.5%. The results of the univariate analysis OR test (Table-1) show that samples collected from wetlands or close to rice fields were 6.1 times (OR: 6.1; 95% CI: 0.71-51.5; p>0.05) more likely to be seropositive for JEV as compared to serum samples collected from dryland fields. The difference in the values was not significant (p>0.05).

**Table 1:** Habitat-related risk factor for Japanese encephalitis seroprevalence in long-tailed macaques.

Location determinants	Number of samples	Seropositive (%)	OR and inverse OR (95% CI)	p-values
Wetland – rice field nearby	84	39 (46.4)	6.1 (0.715-51.5)	p=0.07
Dryland, no rice field	8	1 (12.5)	0.17 (0.02-1.4)	p=0.07

OR=Odds ratio, CI=Confidence interval

Seropositivity of juvenile monkeys (56.6%) was higher than in adults (36.6%). The sera of juvenile monkeys were 2.38 times more likely to be seropositive to JEV than those of adults but the difference was not significant (OR: 2.38, 95% CI: 0.98 - 5.79, p=0.06). The seropositivity of the female monkeys (48.1%) was higher than the male monkeys (36.8%). The female sample sera were 1.79 (OR: 1.79, 95% CI: 0.76 - 4.21; p>0.05) times more likely to have the antibodies against JEV compared to the males (Table-2).

**Table 2:** Animal level determinants associated with JE seroprevalence in long-tailed macaques.

Criteria	Determinants	Number of samples	Seropositive (%)	OR and inverse OR (95% CI)	p-value (mid-p exact)
Age	Juvenile	30	17 (56.6)	2.4 (0.98-5.79)	p>0.05 (p=0.06)
	Adult	62	22 (36.6)	0.4 (0.15-1.12)	p>0.05 (p=0.06)
Sex	Male	38	14 (36.8)	0.56 (0.24-1.32)	p>0.05 (p=0.192)
	Female	54	26 (48.1)	1.79 (0.76-4.21)	p>0.05 (p=0.192)

Juvenile 2-3 years old, adult female>4 years, adult male>6 years. OR=Odds ratio, CI=Confidence interval

In the wetland study site (Ubud), 289 mosquitoes were captured from two light traps over 3 consecutive days. *C. tritaeniorhynchus* and *Anopheles* spp. were the two most abundant mosquito species collected at the wetland study site. Given that the primary vector of JEV is *Culex* spp., only *Culex* mosquitoes were further identified and morphologically differentiated up to the species level. Out of 289 mosquitoes, four species of *Culex* were recorded, wherein 25.3% of the samples was *C. tritaeniorhynchus*, 16.6% of the samples was *C. fuscocephala*, 19.0% of the samples was *C. quinquefasciatus*, and 13.8% of the samples was *C. bitaeniorhynchus*. Finally, 25.3% of samples were identified as *Anopheles* spp. (Table-3). On the other hand, in the dryland study site at Uluwatu temple, 56 mosquitoes were captured for three consecutive days from a total of two light traps (Table-3). The most encountered species at the Uluwatu temple area was *Cx. tritaeniorhynchus* (30.4%).

**Table 3:** The total number of mosquito species captured during three consecutive days at the wetland study site (Ubud) and the dryland study site (Uluwatu).

Mosquitoes	Primary study site				Additional study site			
	
	(Ubud)				(Uluwatu)			
	
	D1	D2	D3	Total	D1	D2	D3	Total
*Culex* spp.								
*Culex tritaeniorhynchus*	30	25	18	73	6	8	3	17
*Culex fuscocephala*	20	15	13	48	8	0	7	15
*Culex quinquefasciatus*	15	30	10	55	2	5	0	7
*Culex bitaeniorhynchus*	5	20	15	40	1	2	0	3
*Anopheles* spp.	25	20	28	73	4	8	2	14
Total	95	110	84	289	21	23	12	56

*Anopheles* spp. not identified and differentiated for their species. Nusa Penida not done due to technical difficulties

## Discussion

This is the first investigation of JE in macaques in Bali. Infection was detected using serological tests. Viral isolation is needed, whether or not the virus develops in monkeys. JE is a vector-mediated, zoonotic disease that is endemic in Indonesia, including the province of Bali [21]. It has also been reported that the disease is a major cause of viral encephalitis in humans, especially in children [22].

Studies on the detection of antibodies against JEV in animals in Bali have mainly focused on farm animals such as pigs, which may be reservoirs or amplifying hosts of the virus. Ducks and chickens are also suspected of having a role in disease transmission [5]. The animals appear to develop viremia to a sufficient titer level to infect feeding mosquitoes [23-25]. The present study was a preliminary investigation into the seroprevalence and determinants of JEV infection in long-tailed macaques. We investigated the seroprevalence of JEV infection in macaques in relation to the ecological profile of wetland areas, in which there may be a high occurrence of JEV infection and a risk of exposure of humans to the virus.

The seroprevalence of JEV antibodies was 43.4%, with 40 macaques positive out of 92 individuals sampled. In the wetland area site, the seroprevalence of the samples was 46.4% (39 positives out of 84 individuals). However, we found only one positive out of eight samples collected from the dryland sites. The high level of detectable JEV antibodies in the samples collected from the primary wetland site suggests a high circulation of the virus in this study area, possibly because of the favorable climatic conditions, which affect the abundance and types of the mosquitoes around the hosts. *C. tritaeniorhynchus* was the main vector of the virus found in this area (Table-3).

*Culex* mosquitoes play a critical role in transmitting JEV from infected animals to susceptible animals and from infected animals to humans. Similar species of mosquitoes were found at both study sites, although in different proportions. In the Uluwatu area, where there is no river or wet rice field, mosquitoes were less abundant than in the wetland study site. *Culex* mosquitoes were also more frequently trapped than other mosquitoes in paddy fields and animal farms. Over the 12 weeks of collection, 46.6% of *Culex* individuals were collected in Badung, and 41.2% were collected in the Tabanan regencies [26]. The JEV needs vectors for its life cycle, and mosquitoes are the primary vectors. Mosquitoes rely on standing water and/or irrigated areas for breeding. *Culex* spp. was abundant in the wetland area and is likely to act as a vector under appropriate environmental conditions [27,28]. Of all *Culex* species captured, *C. tritaeniorhynchus* was the most abundant, possibly because of the environment around the wetland study site. *C. quinquefasciatus* has recently been reported as another potential mosquito vector for JEV in Thailand [29]. Besides, *C. bitaeniorhynchus* and *C. fuscocephala* have also been reported to play a role in local JEV transmission [30]. The presence of viruses in forest areas and their association with birds needs to be further studied, considering the previous report on the contribution of wild and domestic birds [31].

JE is a disease that is closely related to the environment [32]. As previously reported, well-irrigated rice fields are one of the main breeding sites for these mosquitoes [33]. The existence of irrigated rice fields bordering the forest area in the primary site provides an opportunity for the movement of mosquitoes from rice fields to the forest areas. The biological control of mosquitoes to decrease the number of JE cases has been investigated. As chemical mosquito control tends to be harmful, surface feeders and carnivorous fish, aquatic planarians (*Dugesia bengalensis*), beetles (*Acilius sulcatus*), and anuran tadpole prey on mosquito eggs and larvae [34] and may lower the mosquito population and limit JEV transmission.

## Conclusion

This study showed that JEV seropositivity in macaques at the primary study site was high; however, study site needs to be expanded. We estimated the prevalence of mosquito vectors at the study site and predicted their role in local JEV transmission between animals. The study revealed that some populations of long-tailed macaques in Bali had been infected by JEV, with a seroprevalence of 43.4%, and the age and location from which samples were collected influenced the seropositivity. Future studies are required to investigate the role of macaques in maintaining JEV sylvatic cycles in Bali. The long-term dynamics of JEV antibodies and infection (including shedding) in macaques are unknown. Such information is arguably needed before an animal can be designated as a reservoir.

## Authors’ Contributions

IGAAP: Designed the study, collected samples, and drafted the manuscript. AAAMA: Executed the ELISA work, collected the mosquitoes, and drafted the manuscript. IMK: Collected the samples and analyzed the data. INMA: Serological work and revised the manuscript. INW: Coordinate in capturing and handling the monkey and trapping the mosquitoes. IGS: Collected blood sample and prepared sera. FB: Capturing and determining the age and gender of the monkey. AF: Analyzed the data and English corrections. All authors read and approved the final manuscript.
